# A Potential Nervous Necrosis Virus (NNV) Live Vaccine for Sole Obtained by Genomic Modification

**DOI:** 10.3390/ani14060983

**Published:** 2024-03-21

**Authors:** Lucía Vázquez-Salgado, Sandra Souto, José G. Olveira, Isabel Bandín

**Affiliations:** Instituto de Acuicultura, Departamento de Microbiología y Parasitología, Universidade de Santiago de Compostela, 15706 Santiago de Compostela, Spain; sandra.souto@usc.es (S.S.); jose.olveira@usc.es (J.G.O.)

**Keywords:** Betanodavirus, reassortant, NCR, attenuation, live vaccine, survival

## Abstract

**Simple Summary:**

Viral Encephalopathy and Retinopathy (VER) is a severe neurological disease that affects a wide range of fish species, especially at early stages of development. The disease is caused by the Nervous Necrosis Virus (NNV) and is of major global concern as no effective treatments have been described. Therefore, the objective of this study was to obtain an NNV live attenuated vaccine for sole by introducing point mutations in non-coding regions. Vaccination assays were performed on juvenile Senegalese sole by bath, intramuscular, and intraperitoneal injection, at two temperatures (18 and 22 °C); these trials revealed higher survival in vaccinated fish and poorer viral replication, while showing a significant immune response, therefore indicating that the attenuated strain is a good vaccine candidate.

**Abstract:**

Viral Encephalopathy and Retinopathy (VER) is a neurological infectious fish disease that causes vacuolization and necrosis in the central nervous system, which lead to swimming abnormalities and, generally, host death in the early stages of development. VER is caused by the Nervous Necrosis Virus (NNV), a non-enveloped virus with a bisegmented and positive-stranded (+) RNA genome. The largest segment (RNA1) codes for viral polymerase while capsid protein is encoded by RNA2. The aim of this study was to explore the potential of a reverse-engineered RGNNV/SJNNV strain that harbors mutations in both 3′NCRs (position 3073 of RNA1 and 1408 and 1412 of RNA2) as an attenuated live vaccine for sole. The attenuation of this strain was confirmed through experimental infections in sole at 22 °C. Vaccination trials were performed by bath, intramuscular, and intraperitoneal injection, at two temperatures (18 and 22 °C). Our results indicate the improved survival of vaccinated fish and delayed and poorer viral replication, as well as an overexpression of immune response genes linked to T cell markers (*cd4* and *cd8*), to an early inflammatory response (*tlr*7 and *tnfα*), and to antiviral activity (*rtp*3 and *mx*). In conclusion, our study indicates that the attenuated strain is a good vaccine candidate as it favors sole survival upon infection with the wt strain while inducing a significant immune response.

## 1. Introduction

Viral nervous necrosis (VNN) or Viral Encephalopathy and Retinopathy (VER) is a severe disease that affects marine fish worldwide, including farmed species of high commercial value such as grouper (*Epinephelus* spp.), sea bass (*Dicentrarchus labrax*), or sole (*Solea senegalensis*). This disease is especially fatal in early developmental stages, in which infected fish often display neurological signs, such as abnormal swimming behavior, before succumbing to death [1]. The causative agent is the Nervous Necrosis Virus (NNV), a small non-enveloped virus (25–30 nm) belonging to the family *Nodaviridae* (G. *Betanodavirus*). The NNV genome is composed of two molecules of single-stranded and positive sense RNA, known as RNA1 and RNA2 [2]. The RNA1 segment (~3100 bp) contains the open reading frame (ORF) for the viral RNA-dependent RNA-polymerase (RdRp [3]), which drives the synthesis of a subgenomic RNA molecule (RNA3) from the 3′ end of RNA1 during the infection process [2]. The RNA2 molecule (~1400 bp) codes for the capsid protein, the only structural protein carried by the virus [3]. The RNA2 segment holds a conserved sequence, known as the T4 region, that allows for NNV classification into four genotypes: barfin flounder, redspotted grouper, striped jack, and tiger puffer Nervous Necrosis Virus (BFNNV, RGNNV, SJNNV, and TPNNV, respectively [4]). In addition, reassortment between both RNA molecules of the RGNNV and SJNNV genotypes has been described in the Mediterranean area [5,6]. The RGNNV/SJNNV reassortants, which have been demonstrated to be highly pathogenic for sole and gilthead sea bream [7,8,9], contain mutations in the coding and non-coding regions (NCRs) of both RNA molecules with respect to the RGNNV- and SJNNV-type parental strains [6,10]. In 3′NCR, these mutations include positions 3073 and 3093 for RNA1 and 1408 and 1412 for RNA2, which have been demonstrated to be involved in viral replication and virulence [11,12].

Despite the severe consequences of VER episodes in aquaculture hatcheries, effective treatments have not been developed to date, so prevention measures mainly rely on strict biosecurity protocols. Several attempts to develop betanodavirus vaccines have been assayed, including inactivated, subunit, DNA, and live vaccines [13,14,15,16,17,18]. Live attenuated strains obtained by genetic modification are promising candidates for vaccine development, as the immune response they elicit reproduces the response triggered during an infection with wild strains and thereby provides long-term protection. Additionally, vaccination requires lower doses that are cheaper and easier to produce than other vaccines and that have a low impact on the environment [19,20]. In the present study, we have explored the potential of a reverse-engineered NNV strain with mutations in the 3′NCR of both genomic segments as a live attenuated vaccine, focusing on its in vivo performance in terms of fish survival, viral replication, and immune response.

## 2. Materials and Methods

### 2.1. Attenuation and Propagation of Viral Strains

**Figure 1:**
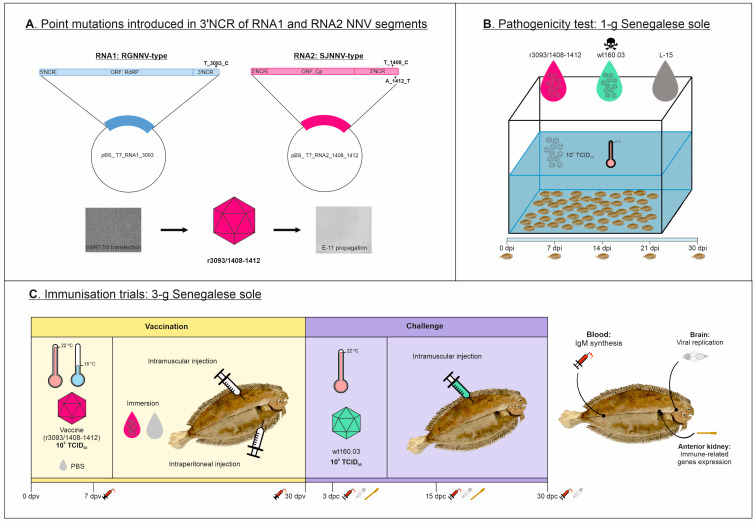
Experimental design of this study. A recombinant NNV strain harboring point mutations in 3′NCR of RNA1 and RNA2 segments was obtained by reverse genetics (**A**). Attenuation of this strain was evaluated through bath infection of 1 g sole at 22 °C (**B**), and vaccination trials were performed using 3 g sole juveniles (**C**) through intramuscular (im) and intraperitoneal (ip) injection and immersion at 18 and 22 °C, and survivors were challenged with the wt strain. Samples were collected at 7 and 30 dpv, and at 3, 15, and 30 dpc, for virological and immunological analyses.

The reassortant RGNNV/SJNNV strain IAuscSpSs160.03 [6], a hypervirulent strain for sole, hereafter wt160.03, was used as the reference wild-type (wt) strain in this study. A recombinant viral strain was obtained by reverse genetics (Figure 1A) using two plasmids that contain the full-length cDNA of both RNA molecules with point mutations in the 3′-NCR regions of RNA1, at position 3093 (T → C), and RNA2, at positions 1408 (T → C) and 1412 (A → T). Plasmids, namely R1_r3093 and R2_r1408-1412 (1 µg each), were transfected in BSRT7/5 cells following the protocol described by Souto et al. [10]. The obtained recombinant virus, hereafter r3093/1408-1412, and strain wt160.03 were propagated in E-11 cell line monolayers using Leibovitz’s 15 medium (L-15; Gibco) supplemented with 5% fetal bovine serum (FBS; Corning). Infected cells were incubated at 25 °C until cytopathic effect was complete. Then, crude virus was clarified (3000× *g* for 20 min) and subjected to viral titration by inoculating ten-fold dilutions in E-11 monolayers seeded in 96-well plates. Viral titer was estimated according to the Reed and Muench procedure as 50% tissue culture infective dose (TCID_50_ mL^−1^) [21].

The genomic stability of the point mutations introduced in the 3′NCR of both genomic segments was confirmed after 10 serial passages in E-11 cells. The complete 3′-NCR sequence of the obtained virus was determined through RACE using a FirstChoice RLM-RACE kit (Ambion; Thermo Fisher Scientific, Madrid, Spain) as described by Souto et al. [10].

### 2.2. In Vitro Replication

The recombinant r3093/1408-1412 and wt160.03 were inoculated at an MOI of 0.1 in triplicate in E-11 cells seeded in 48-well plates. After 1 h of adsorption, monolayers were washed three times with L-15 medium and incubated with L-15 supplemented with 2% FBS for 7 days at 25 °C. Samples (100 µL) were collected 24, 48, 72, 144, and 168 h post infection (hpi) and stored at −20 °C for virological analysis by titration and RNA quantification.

### 2.3. Senegalese Sole Infection Challenges

Senegalese sole (mean body weight 1 ± 0.1 g and 3 ± 0.1 g) were acclimated in opaque 300-L tanks containing recirculating sea water (salinity 33 g L^−1^) at 18 °C and fed ad libitum at the facilities of the University of Santiago de Compostela. Fish were strictly handled according to the current regulations on animal welfare (Directive 2010/63/UE) in experimental procedures, and the experimental protocol was approved by the Bioethics and Experimental Animal Welfare Committees of the University of Santiago de Compostela and Xunta de Galicia (Permit Id. 15010/2020/004). Prior to each challenge, 10 individuals per fish size were randomly collected and euthanized using an MS-222 (Sigma-Aldrich; St. Louis, MI, USA) overdose and tested for the presence of bacterial and viral pathogens. Bacterial analyses were accomplished by kidney, spleen, and liver inoculation onto tryptone soy agar supplemented with 1% NaCl (TSA-1) and incubated at 25 °C for 24 h. For virological analyses, real time quantitative PCR (RT-qPCR) was performed using specific primer sets for NNV, viral hemorrhagic septicemia virus (VHSV), infectious pancreatic necrosis virus (IPNV), and infectious hematopoietic necrosis virus (IHNV) [22]. For the experimental infections, water temperature was maintained at 18 °C or increased by 0.5 °C per day up to 22 °C, depending on the conditions of each trial.

#### 2.3.1. Pathogenicity Test

Senegalese sole early juveniles (1 g) were bath-infected with r3093/1408-1412 and wt160.03 strains (*n* = 90 each) at a concentration of 10^5^ TCID_50_ mL^−1^ (Figure 1B). After a 3 h immersion at 22 °C, fish were randomly divided into 3 replicate tanks per condition. Also, a group (*n* = 30) of mock-infected fish (treated with L-15) was set up and handled as the infected groups. Fish were supervised daily to remove dead fish and to check for the appearance of clinical signs. A sample of 3 individuals was randomly collected on a weekly basis from one replicate tank per infection group for virological analyses until the experiment was finished on day 30 post infection (pi).

The head region of the sampled fish was aseptically removed and individually processed according to the procedure detailed by Olveira et al. [6]. Sample supernatants were subjected to viral titration as previously described and to RNA extraction.

#### 2.3.2. Immunization Trials by Different Routes and at Two Temperatures

Senegalese sole early juveniles (3 g) were vaccinated with r3093/1408-1412 (10^5^ TCID_50_ fish^−1^) at 18 and 22 °C by bath immersion and intramuscular (im) injection (Figure 1C). In addition, for comparative purposes, intraperitoneal (ip) injections were also performed at 22 °C. Control fish were mock-vaccinated with PBS (100 µL) and handled as the infected groups. Three replicate tanks (*n* = 100) were established per condition and were supervised daily to collect dead fish and monitor for clinical signs. On day 30 post vaccination (pv), surviving fish from vaccinated and mock-vaccinated groups were challenged by im injection with the wt160.03 strain (10^5^ TCID_50_ fish^−1^) and reared for a further 30 days at 22 °C. 

Virological analyses (RNA2 quantification) were performed in vaccinated and mock-vaccinated groups 3, 15, and 30 days post challenge (dpc). Humoral immune response (anti-NNV IgM) was assessed in sera of vaccinated and control fish 7 and 30 dpi and 3, 15, and 30 dpc, while immune-related gene expression was only evaluated in fish vaccinated through im at 22 °C, on days 7 and 30 pv and 3 and 15 post challenge (pc). Samples (*n* = 6) were randomly collected from one replicate tank per condition at each respective time point, and brains and anterior kidneys were aseptically removed and subjected directly to RNA extraction.

### 2.4. Reverse-Transcription Real-Time Quantitative PCR (RT-PCR)

Total RNA extraction was accomplished using a Nucleospin ^®^ Kit (Macherey-Nagel; Duren, Germany) according to the manufacturer’s guide. Complementary DNA (cDNA) synthesis was performed by RNA reverse transcription in a thermocycler MyCycler™ (Bio-Rad; Hercules, CA, USA), through 5 min incubation at 95 °C with 200 nM of random primers and then for 1 h at 42 °C with the reverse transcription mixture containing a RevertAid First Strand cDNA Synthesis Kit (Thermoscientific Inc.; Vilnius, Lithuania). The protocol was finished with enzyme inactivation through 10 min incubation at 70 °C.

NNV quantification (RNA1 and RNA2) was performed by RT-qPCR in a CFX-96 Real-Time PCR detection system (Bio-Rad). Briefly, cDNA was amplified with 200 nM of SnodR1 F/R or NodR2 F/R primers [23], when applicable, and BlasTaq™ 2 × qPCR Master Mix (abm), following the manufacturer’s instructions except for the annealing extension step (20 s at 59 °C for SnodR1 and at 57 °C for NodR2). The total RNA amount expressed as RNA1 or RNA2 copies g^−1^ was extrapolated from two standard curves consisting of 20-fold dilutions of two plasmids containing the full-length cDNA of RNA1 and RNA2 molecules of the wt160.03 strain (RNA1: 2.91 × 10^7^ copies µL^−1^; RNA2: 4.22 × 10^7^ copies µL^−1^).

### 2.5. Immunological Analyses

#### 2.5.1. Specific IgM Production

IgM production was estimated in the sera of vaccinated and mock-vaccinated 3 g fish. For this purpose, blood samples were collected from the caudal peduncle of sole and incubated for 4 h at 4 °C. The sera fraction was separated by centrifugation at 10,000× *g* for 10 min, and the quantification of specific anti-NNV IgM was estimated according to the protocol described by Valero et al. [24].

#### 2.5.2. Immune-Related Genes Expression

The expression of *cd*4, *cd*8, *mx*, *rtp*3, *tlr*7, and *tnf*α genes was assessed in the anterior kidney of 3 g sole vaccinated through im injection and reared at 22 °C, 7 and 30 days post vaccination (dpv) and 3 and 15 dpc. To this end, the cDNAs obtained above were subjected to RT-qPCR as previously described, using the specific primer set and annealing-extension conditions described by [17,24,25] and listed in Table 1. The transcription of each gene was estimated by the 2^−ΔΔCt^ method [26], with the endogenous ß-actin (ß-*act*) gene as a reference and mock-vaccinated fish as the control.

### 2.6. Statistics

The software used for data analyses was GraphPad Prism 8.1. Survival curves were compared using the Kaplan–Meier test and differences were calculated by the log-rank (Mantel–Cox) test. Data describing viral replication and immune response are expressed as the mean ± SD of the biological replicates in each time point. Data were tested for normality (Shapiro–Wilk test) and then analyzed by two-way ANOVA with Tukey and Dunnett’s correction. A *p* value < 0.05 represents statistically significant differences. 

Relative Percentage Survival (RPS) was determined according to the following formula: RPS = 1 − [% mortality vaccinated fish/% mortality control fish] × 100.

## 3. Results

### 3.1. Growth Kinetics in E-11

**Figure 2:**
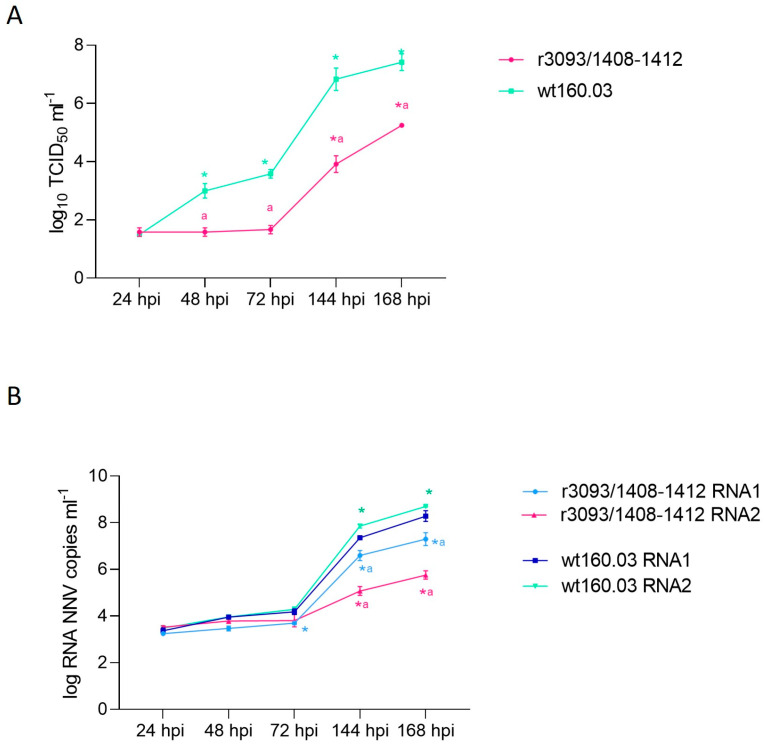
In vitro replication of the recombinant r3093/1408-1412 in E-11 cells at 25 °C. RNA1 and RNA2 load, expressed as log copy number mL ^−1^ (**A**), and progeny production, expressed as log TCID_50_ mL^−1^, at 24, 48, 72, 144, and 186 hpi (**B**). Asterisks (*) indicate statistically significant differences regarding 24 hpi, and the letter a represents statistically significant differences regarding wt160.03 data at the same time point (*p* value < 0.05).

The recombinant r3093/1408-1412 showed delayed growth when compared with the wt strain (Figure 2A). Up to 72 hpi, the titer of the recombinant strain showed no increase (10^1.75^ TCID_50_) and showed a significant difference with the wt strain (*p* < 0.05). Although, from 72 hpi onwards, recombinant replication was indeed recorded (10^3.92^ and 10^5.25^ TCID_50_ mL^−1^, at 144 and 168 hpi, respectively), it occurred slower and to a lower extent than in the wt160.03 (10^6.83^ and 10^7.14^ TCID_50_ mL^−1^, respectively). Although RNA replication was quite similar in both the wt and recombinant strains until 72 hpi (Figure 2B), significant differences were detected from that time point onwards. Thus, at 168 hpi, the RNA 1 and RNA 2 load (10^7.29^ and 10^5.75^ copies mL^−1^, respectively) was around 1 and 3 logs lower than that recorded in the wt strain (10^8.28^ and 10^8.68^, respectively). In addition, whereas no significant difference was observed in the production of both RNA molecules in the wt strain, in the recombinant strain, the synthesis of RNA2 was 1.3-fold lower than that of RNA1.

### 3.2. Pathogenicity Test

**Figure 3:**
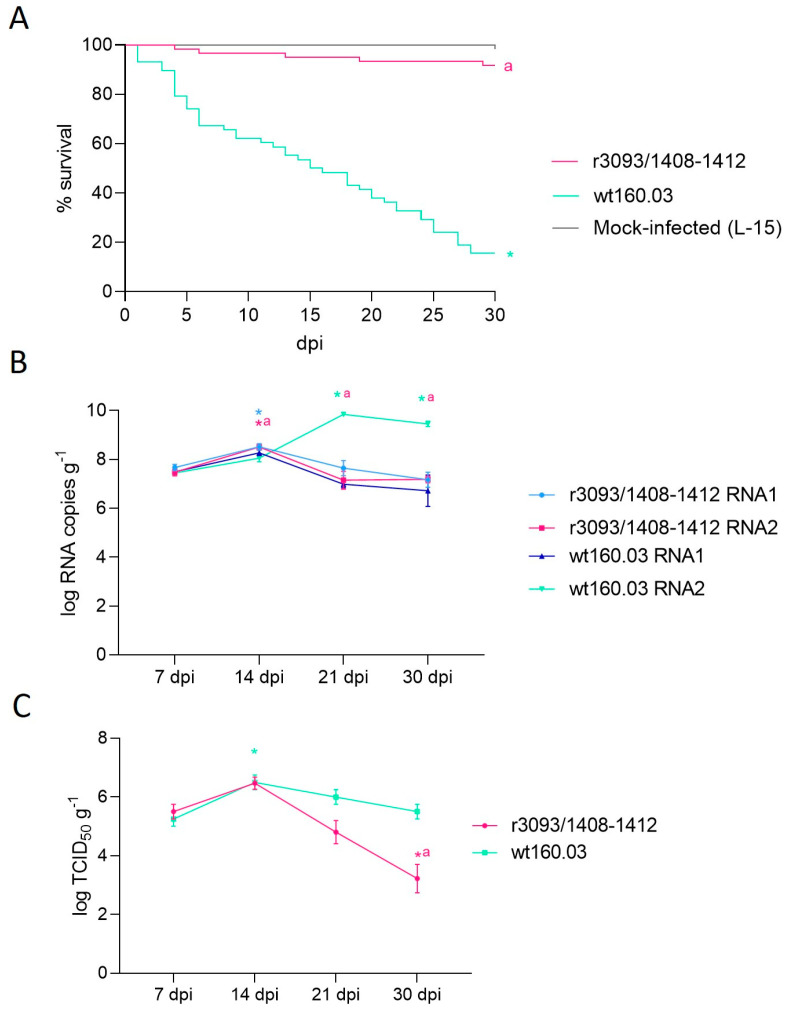
Pathogenicity test conducted using one-gram Senegalese sole subjected to bath infection at 22 °C. Survival curves expressed as percentage of survival (%) during 30 days of infection with strains r3093/1408-1412 and wt160.03 and a mock-infected group with L-15 (**A**). RNA1 and RNA2 load on days 7, 14, 21, and 30 pi, expressed as log RNA copies g^−1^, in r3093/1408-1412 and wt160.03-infected sole (**B**). Quantification of NNV infective particles in sole brain 7, 14, 21, and 30 pi, expressed as TCID_50_ mL^−1^ (**C**). Asterisks (*) indicate statistically significant differences regarding mock-infected fish (**A**) or 7 dpi (**B**,**C**), and letter a represents statistically significant differences regarding wt160.03 data at the same time point (*p* value < 0.05).

The attenuation of the r3093/1408-1412 strain was evaluated in 1 g sole juveniles by bath immersion at 22 °C using the wt strain as control. Although abnormal swimming behavior was displayed to a similar extent in both groups of infected fish, attenuation was confirmed by the survival of 92.23% of the individuals inoculated with the recombinant, while only 15.56% of fish survived the infection with the wt strain (Figure 3A). The difference between survival curves was statistically significant (log-rank (Mantel–Cox) test < 0.0001).

NNV replication in fish was expressed as the number of RNA1 and RNA2 copies g^−1^ (Figure 3B) and as TCID_50_ g^−1^ (Figure 3C). A similar viral load was detected 7 dpi (on average, 10^7.5^ RNA copies and 10^5.5^ TCID_50_ g^−1^) in sole infected with r3093/1408-1412 and the wt, and then 14 dpi, both strains showed a ~1-log increase. However, afterwards, a reduction in RNA1 copies was detected (down to 10^7.1^ and 10^6.7^ for the recombinant and the wt strain, respectively), whereas a clear increase in RNA2 copies was observed only in the wt strain (*p* value < 0.05, Figure 3B). The recovery of viable particles in cell culture also showed a significant difference between both strains at the end of the experimental period (Figure 3C). Thus, on day 30 pi, fish infected with the recombinant virus contained 10^3.2^ TCID_50_ g^−1^, 2 logs lower than the value recorded in fish infected with the wt strain (10^5.5^ TCID_50_ g^−1^; *p* value = 0.02).

### 3.3. Immunization Trials

#### 3.3.1. Survival Curves and Relative Percent Survival (RPS)

The potential of the recombinant strain as a live vaccine was assessed in 3 g sole at 18 and 22 °C by different routes. Challenges were performed in all cases by im to ensure the administration of the same viral dose to each fish.

**Figure 4:**
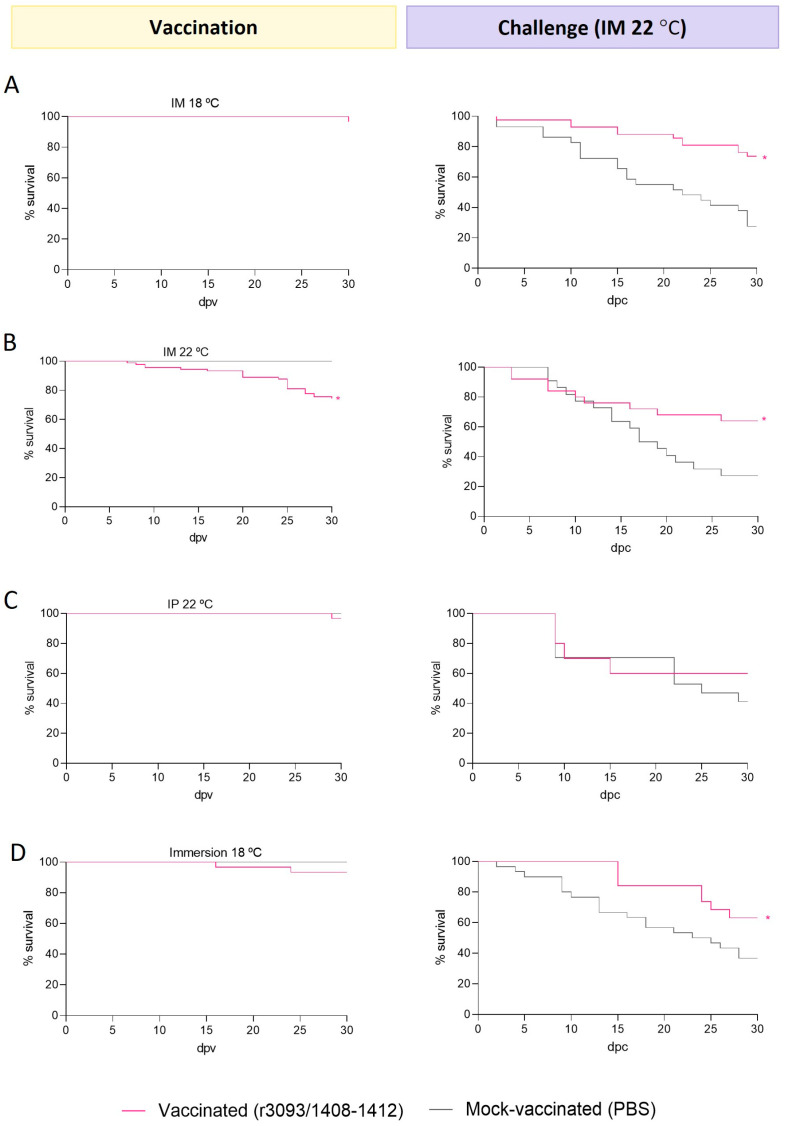
Immunization trials in 3 g sole. Survival curves (%) upon vaccination through im injection at 18 °C (**A**) and 22 °C (**B**), ip (**C**), and immersion (**D**), and after challenge with wt. Asterisks (*) indicate statistically significant differences regarding mock-vaccinated fish (*p* value < 0.05).

- Intramuscular injection: Vaccination at 18 °C caused low mortality (96% survival; Figure 4A), whereas slightly lower survival (74%) was observed at 22 °C (Figure 4B). After the challenge, the survival of fish vaccinated at 18 and 22 °C was 74 and 64%, respectively, which is significantly higher than that recorded in the mock-vaccinated group (27% at both temperatures; *p* value < 0.0001). Thus, at both temperatures, RPS values were above 50, but the highest value was obtained in fish vaccinated at 18 °C (64 vs. 51).
- Intraperitoneal injection at 22 °C: Low mortalities were recorded in vaccinated fish (96% survival; Figure 4C) during the immunization period. However, survival was not improved after the challenge, since no significant differences were observed with respect to the control group.
- Immersion: The survival of fish vaccinated at 18 °C was 93% (Figure 4D) and, after the challenge, it was significantly improved (63%) compared to mock-vaccinated groups (36%; *p* value < 0.05), which represents an RPS of 42%. Unfortunately, unexpected mass mortalities were detected in fish mock-vaccinated at 22 °C during the immunization period, so this condition was not considered in the study.

#### 3.3.2. RNA2 Quantification after the Challenge

**Figure 5:**
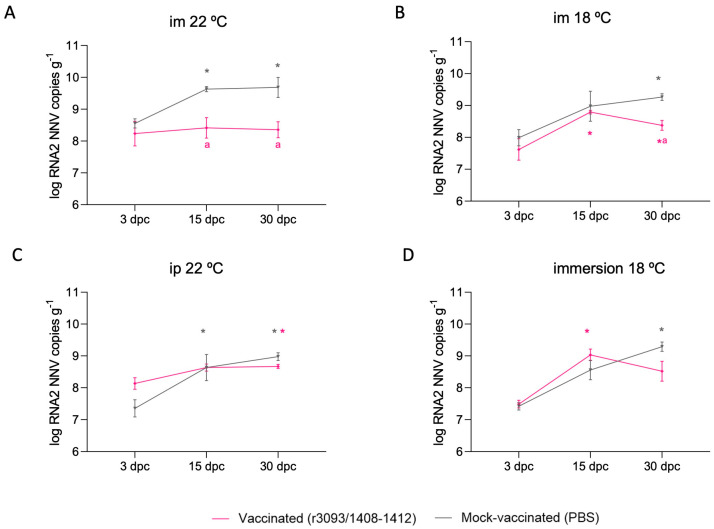
RNA2 genomic copies quantified 3, 15, and 30 dpc in surviving fish from the immunization period and subjected to im-challenge at 22 °C. RNA2 load in fish previously inoculated through im at 22 °C (**A**) and at 18 °C (**B**), by ip (**C**), and by immersion (**D**). Asterisks (*) indicate statistically significant differences regarding 3 dpc, and the letter a indicates the same regarding mock-vaccinated fish at the same time point (*p* value < 0.05).

The RNA2 load was quantified 3, 15, and 30 dpc in vaccinated and control fish. The amount of total RNA2 copies in fish vaccinated by im injection at 22 °C was 1.32 logs lower on day 30 pc than in mock-vaccinated fish (Figure 5A; *p* value < 0.0001), whereas at 18 °C it was 0.88 logs lower (Figure 5B). This difference was reduced in fish vaccinated through immersion and via ip injection (0.77 and 0.31 logs lower, respectively; Figure 5C,D).

#### 3.3.3. Specific Anti-NNV IgM Production 

**Figure 6:**
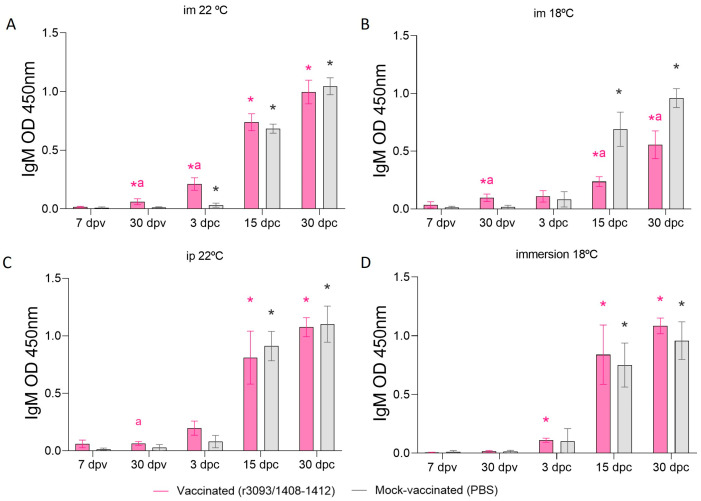
Humoral immune response, expressed as IgM OD 450 nm, assessed in fish sera of vaccinated and mock-vaccinated fish. Specific anti-NNV IgM detected in fish vaccinated through im injection at 22 °C (**A**) and at 18 °C (**B**), by immersion at 18 °C (**C**) and by ip injection (**D**). Asterisks (*) indicate statistically significant differences regarding 7 dpv, and the letter a indicates statistically significant differences regarding mock-vaccinated fish at the same time point (*p* value < 0.05).

Humoral immune response was assessed in the sera of vaccinated and mock-vaccinated fish through im injection at 22 (Figure 6A) and 18 °C (Figure 6B), ip injection at 22 °C (Figure 6C), and immersion at 18 °C (Figure 6D) on days 7 and 30 pv, as well as after the challenge (3, 15, and 30 dpc). At the end of the immunization period, fish vaccinated by im and ip injection revealed significant IgM production (*p* value > 0.05) that was not observed in the fish vaccinated by immersion. After the challenge (3 dpc), fish vaccinated through im injection at 22 °C showed a significantly higher IgM synthesis than control fish (OD 450 nm: 0.21 vs. 0.03; *p* value < 0.01), while vaccination at 18 °C caused significantly lower production on days 15 and 30 pc (OD 450 nm: 0.23 and 0.55, respectively) than mock-vaccinated groups (OD 450 nm: 0.69 and 0.96, respectively; *p* value < 0.05). Vaccination through immersion or ip injection induced similar IgM synthesis after the challenge as in the control groups (*p* value > 0.05).

#### 3.3.4. Immune-Related Genes Expression in Brain and Anterior Kidney of Im-Infected Fish at 22 °C

**Figure 7:**
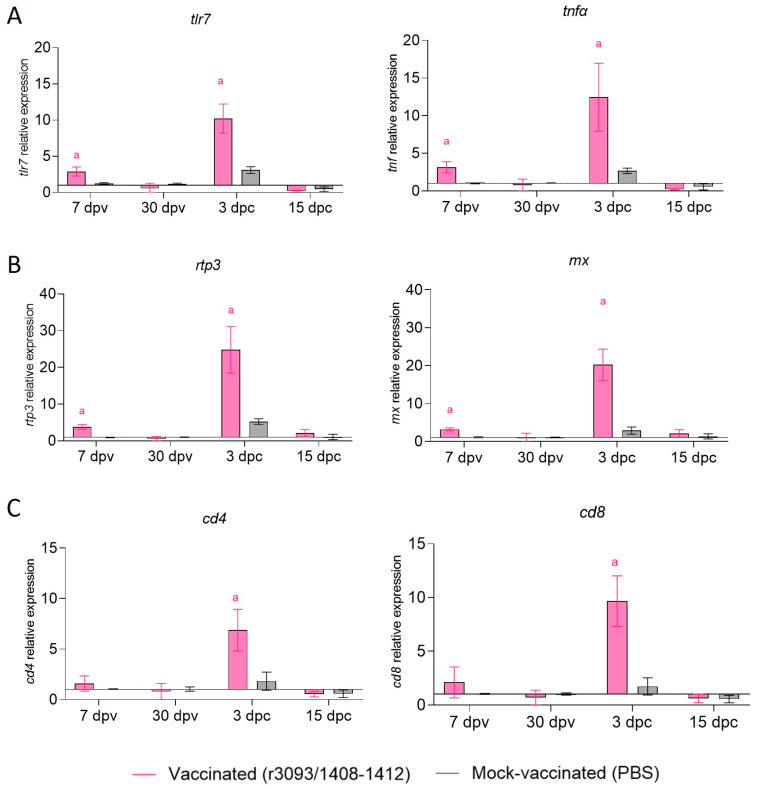
Relative expression of immune-related genes in anterior kidney of vaccinated and mock- vaccinated fish through im injection at 22 °C. Early inflammatory response genes (*tlr7* and *tnfα*; (**A**)), antiviral genes (*rtp3* and *mx*; (**B**)), and cellular response genes (*cd4* and *cd8*; (**C**)). Letter a indicates statistically significant differences regarding mock-vaccinated fish at the same time point (*p* value < 0.05).

The transcription of immune-related genes was only evaluated in the anterior kidney of fish vaccinated through im at 22 °C, both after vaccination and challenge (Figure 7). The expression of genes putatively related with an inflammatory response (*tlr7* and *tnfα*; Figure 7A) and antiviral protein-coding genes (*rtp3* and *mx*; Figure 7B) was significantly up-regulated on day 7 pv, while no changes were observed in cellular response (*cd4* and *cd8*; Figure 7C). After the challenge, all assessed genes were overexpressed regarding mock-vaccinated fish, although only on day 3 pc.

## 4. Discussion

The development of an effective vaccine against NNV to prevent or reduce the severity of VER episodes on fish farms is still a challenge for researchers and global aquaculture. In recent years, numerous experimental vaccines have been tested, but only two inactivated vaccines protecting against RGNNV infection in sea bass are commercially available [1]. In this work, we have evaluated an attenuated NNV mutant as a live vaccine candidate to prevent VER infections in sole.

Previous studies indicated that NNV reassortant strains isolated from sole show differences in the 3′ NCR of both genomic segments with respect to the parental strains [10] and that the reversion of some of these changes in each NCR led to partial attenuation [11,12]. Therefore, with the aim of achieving a fully attenuated strain, we constructed the recombinant r3093/1408-1412 (indicating the mutated positions in the 3′NCR of the RNA1 and RNA2 segments, respectively). The attenuation of this recombinant was evaluated by immersion, the method that better resembles natural infections, and using 1 g sole, a fish size highly susceptible to NNV [8]. Infection with the recombinant mimicked the clinical signs of infection with the wt but caused very low mortality (92.23% survival vs. 15.56%). Moreover, its replication in sole brain was clearly lower (more than 2 logs TCID_50_) at the end of the experimental infection, in agreement with the slower and lower replication observed in E-11 cells. Although the survival value was similar to that recorded in fish infected with recombinant strains harboring mutations in only one genomic segment (r3093: 70%; r1408-1412: 75% [11,12]), the delayed replication of r3093/1408-1412 when compared with the wt strain was twice that observed in the r1408-1412 mutant [11]. Interestingly, the quantification of both genomic segments indicated that the RNA1 load in sole brain was similar regardless of the viral strain, but the number of RNA2 copies in fish infected with the recombinant was significantly lower from day 14 pi onwards (2.26 log RNA2 copies 30 dpi). These results support a previous study which suggested that mutations in positions 1408 and 1412 reduce RNA2 synthesis and hinder the interaction of this RNA molecule with host cell proteins [11], but they also point to a relevant role in NNV replication of RNA1 3′NCR, especially position 3093. In fact, it is known that the genomic replication of nodavirus depends on cis-acting elements at the ends of both RNA molecules [29]. Alternatively, the change in position 3093 could have altered the synthesis of RNA3, a subgenomic segment synthesized from the 3′ end of RNA1 during NNV infection [30], reported to act as a transactivator of RNA2 replication [31].

Experimental vaccination assays were performed using 3 g sole, in order to be able to test injection vaccination. The vaccination assays were performed at two temperatures: 22 °C, because this is the optimal temperature for the in vivo replication of reassortant NNV strains [8], and 18 °C, the temperature at which fish are commonly reared on the hatcheries in our area. The most promising results were obtained in fish vaccinated by im injection at 18 °C (RPS 64%) and at 22 °C (RPS 51%), which were supported by a significantly lower viral load in fish brains (*p* < 0.05). Immunization with live NNV strains at a suboptimal temperature for viral replication has already been reported to confer high protection levels against re-infection in sevenband grouper (*Epinephelus septemfasciatus*), which was correlated with a very low viral load in fish brains [32,33]. However, in our study, although the RPS value was slightly higher at 18 than at 22 °C, a similar viral load was recorded in the brain of infected individuals maintained at both temperatures. This could be due to the different fish sizes used (more than 50 g vs. 3 g in our assay), the °C difference between the suboptimal and optimal temperature (9 vs. 4), and the immune induction period that, in our study, was only 1 month because of the capacity limitations of our aquarium facilities that do not allow us to extend vaccination trials for a long period. 

Vaccination through immersion at 18 °C provided an RPS of 42%, although only a slight reduction in NNV load in sole brain was observed. Immersion is the best option to vaccinate small-sized fish, like those affected by NNV, because it requires less handling. This vaccination procedure stimulates mucosal immunity, but it is not possible to ensure the uptake of a standardized dose by all individuals, and a systemic immune response may not be achieved [34,35]. Injection via im or ip allows the administration of the same dose to each fish, provides longer protection [20], and allows NNV to spread from the injection site through nervous cells to the brain. However, the virus inoculated by ip injection can be cleared or inactivated by a nonspecific immune response triggered in the peritoneal cavity [36], which could explain the poor results obtained when the attenuated vaccine was administered by this route.

The analysis of the immune response triggered by the vaccine indicated that im injection at 22 °C induced IgM production at the end of the immunization period. However, after the challenge, the immune response was similar to that of the control groups. In contrast, a puzzlingly low amount of anti-NNV IgM was detected in fish vaccinated at 18 °C, although the recombinant replicates much the same as at 22 °C. The scarce role of humoral immunity in protection against NNV infection in sole has already been reported [17,24]; therefore, the role of other immune response players must be considered. The induction of an inflammatory response is critical to the efficiency of the immune adaptative response to viral infection [37]. The live vaccine seems to trigger an appropriate inflammatory response, as indicated by the up-regulation 7 dpv of the *tlr7* and *tnfα* genes. Among Toll-like receptors, TLR-7 detects single-stranded RNA like the NNV genome [38] and stimulates tissue-resident macrophages to produce pro-inflammatory cytokines, including TNFα [39]. TNFα is one of the earliest immune genes expressed in infected fish as it is involved in triggering the expression of other genes associated with the inflammation process [40]. Vaccination also induced the up-regulation of genes coding for the antiviral proteins Mx and RTP3. However, the expression of *cd4* (T helper cells marker) and *cd8* (cytotoxic T lymphocytes) genes was not significantly modulated. After the challenge at 3 dpc, transcription of all immune-related genes was recorded in the vaccinated fish to a much greater extent than in mock-vaccinated fish, reflecting the differences between the adaptative and innate immune system. Fish CD4+ T-helper cells are thought to coordinate the immune response, and cytotoxic T lymphocytes (CTLs) play a major role in antiviral immunity [41,42]. After the challenge, significant up-regulation of CD4 and the CTL marker CD8 was recorded when compared with the mock-vaccinated fish. These data suggest that although neither *cd4* nor *cd8* modulation was recorded after vaccination at the sample points considered in this study, the vaccine does stimulate the cellular response. On the other hand, the up-regulation of genes related with the inflammatory response was much higher after the challenge than after vaccination. The overexpression of inflammatory genes has been associated with an acute phase reaction which caused the sudden death of some NNV carrier sole individuals after being injected with an NNV-inactivated vaccine [25]. However, such a correlation did not seem to occur in our study, since on day 3 pc, only two fish died, and no additional mortalities were recorded until day 7 pc. In spite of this, cytokine overproduction may lead to a homeostatic imbalance in the kidney, eventually leading to a decrease in the immune response [43], as observed 15 dpc. The down-regulation of a variety of immune-related genes several weeks after the challenge seems to be a common feature in NNV vaccination assays performed in sole and also in grouper [18,24,25,44]. Alternatively, this reduction could be related to mechanisms triggered by the virus to evade the host immune system [45].

## 5. Conclusions

The overall results of this study suggest that the recombinant strain r3093/1408-1412 can be considered a suitable live attenuated vaccine candidate for sole if administered through intramuscular injection at 22 or 18 °C, although attenuation should be further increased. The most straightforward option is to include the change in position 3073 of RNA1, as lower mortality was also achieved in fish infected with the mutant strain harboring this change [10], but additional mutations at 3′ NCR of both genomic segments should also be considered. Although some candidates for live attenuated fish vaccines against IHNV, infectious spleen and kidney necrosis virus (ISKNV), and VHSV have been designed by gene deletion or rearrangement [18,46,47,48], this strategy cannot be applied to Betanodavirus because each RNA segment contains a unique ORF. Another approach to improve fish survival upon vaccination with the recombinant strain could be to increase the immunization period to allow the fish to clear the virus or to use a lower viral titer for the vaccination. Both options will be considered in future studies.

## Figures and Tables

**Table 1:** List of the primers sequence used for gene expression analysis.

Gene	Primers Sequence (5′-3′)	Amplicon Size (bp)	Accesion No (a), or Unigene ID (b)
*cd*4	F: GACCTCAGGCTGCAATGGT R: TGAGCAGAGTGATGGACAGACT	65	solea_v4.1_unigene450963 [27]
*cd*8	F: GTCGCAGTTCTGCTCTCCGC R: TCGGTTGCAGTAGAGGACGG	97	solea_v4.1_unigene59609
*mx*	F: CCTCTCTCCTTCAGGATCCTCCTCCTGTGC R: CAAAACAAGAAACTATCTGCCTGGTGGTTC	104	AY790537 [28]
*rtp*3	F: GACGCCCCAATGGTGGAT R: CCAGATTCTTCATGAGGATGGTGAT	64	XM_044043478 [28]
*tlr*7	F: GGGAGTGAGGTCAAAGTGGA R: CGTGGAAGGAGGAGGAGTTT	130	XM_044052250 [25]
*tnf*α	F: TGTGTACATGGGAGCTGTGT R: CACAGAGCGAACACACCAAA	126	XM_044052089 [25]
ß-*act*	F: GACGACATGGAGAAGATC R: GGTGTTGAAGGTCTCAAA	150	DQ485686 [17,24,25]

(a) https://www.ncbi.nlm.nih.gov/nuccore (accessed on 27 February 2024); (b) https://www.scbi.uma.es/soleadb (accessed on 27 February 2024).

## Data Availability

Data are contained within the article.

## References

[1] Bandín I., Souto S. (2020). Betanodavirus and VER Disease: A 30-Year Research Review. Pathogens.

[2] Nagai T., Nishizawa T. (1999). Sequence of the Non-Structural Protein Gene Encoded by RNA1 of Striped Jack Nervous Necrosis Virus. J. Gen. Virol..

[3] Mori K.I., Nakai T., Muroga K., Arimoto M., Mushiake K., Furusawa I. (1992). Properties of a New Virus Belonging to Nodaviridae Found in Larval Striped Jack (*Pseudocaranx dentex*) with Nervous Necrosis. Virology.

[4] Nishizawa T., Furuhashi M., Nagai T., Nakai T., Muroga K. (1997). Genomic Classification of Fish Nodaviruses by Molecular Phylogenetic Analysis of the Coat Protein Gene. Appl. Environ. Microbiol..

[5] Toffolo V., Negrisolo E., Maltese C., Bovo G., Belvedere P., Colombo L., Valle L.D. (2007). Phylogeny of Betanodaviruses and Molecular Evolution of Their RNA Polymerase and Coat Proteins. Mol. Phylogenet. Evol..

[6] Olveira J.G., Souto S., Dopazo C.P., Thiéry R., Barja J.L., Bandín I. (2009). Comparative Analysis of Both Genomic Segments of Betanodaviruses Isolated from Epizootic Outbreaks in Farmed Fish Species Provides Evidence for Genetic Reassortment. J. Gen. Virol..

[7] Souto S., Lopez-Jimena B., Alonso M.C., García-Rosado E., Bandín I. (2015). Experimental Susceptibility of European Sea Bass and Senegalese Sole to Different Betanodavirus Isolates. Vet. Microbiol..

[8] Souto S., Olveira J.G., Bandín I. (2015). Influence of Temperature on Betanodavirus Infection in Senegalese Sole (*Solea senegalensis*). Vet. Microbiol..

[9] Toffan A., Pascoli F., Pretto T., Panzarin V., Abbadi M., Buratin A., Quartesan R., Gijón D., Padrós F. (2017). Viral Nervous Necrosis in Gilthead Sea Bream (*Sparus aurata*) Caused by Reassortant Betanodavirus RGNNV/SJNNV: An Emerging Threat for Mediterranean Aquaculture. Sci. Rep..

[10] Souto S., Biacchesi S., Olveira J.G., Mérour E., Brémont M., Bandín I. (2015). In Vitro and in Vivo Characterization of Molecular Determinants of Virulence in Reassortant Betanodavirus. J. Gen. Virol..

[11] Souto S., Olveira J.G., Dopazo C.P., Borrego J.J., Bandín I. (2018). Modification of Betanodavirus Virulence by Substitutions in the 3’ Terminal Region of RNA2. J. Gen. Virol..

[12] Gémez-Mata J., Souto S., Bandín I., Alonso M.D.C., Borrego J.J., Labella A.M., García-Rosado E. (2021). Immune Response of Senegalese Sole against Betanodavirus Mutants with Modified Virulence. Pathogens.

[13] Valero Y., Awad E., Buonocore F., Arizcun M., Esteban M.Á., Meseguer J., Chaves-Pozo E., Cuesta A. (2016). An Oral Chitosan DNA Vaccine against Nodavirus Improves Transcription of Cell-Mediated Cytotoxicity and Interferon Genes in the European Sea Bass Juveniles Gut and Survival upon Infection. Dev. Comp. Immunol..

[14] Hazreen-Nita M., Azila A., Mukai Y., Firdaus-Nawi M., Nur-Nazifah M. (2019). A Review of Betanodavirus Vaccination as Preventive Strategy to Viral Nervous Necrosis (VNN) Disease in Grouper. Aquac. Int..

[15] Barsøe S., Toffan A., Pascoli F., Stratmann A., Pretto T., Marsella A., Er-Rafik M., Vendramin N., Olesen N.J., Sepúlveda D. (2021). Long-Term Protection and Serologic Response of European Sea Bass Vaccinated with a Betanodavirus Virus-like Particle Produced in *Pichia pastoris*. Vaccines.

[16] Lei Y., Xiong Y., Tao D., Wang T., Chen T., Du X., Cao G., Tu J., Dai J. (2022). Construction of Attenuated Strains for Red-Spotted Grouper Nervous Necrosis Virus (RGNNV) via Reverse Genetic System. Viruses.

[17] López-Vázquez C., Souto S., Olveira J.G., Riaza A., González Ó., Brea C., Labella A.M., Castro D., Bandín I. (2023). Nervous Necrosis Virus (NNV) Booster Vaccination Increases Senegalese Sole Survival and Enhances Immunoprotection. Animals.

[18] Souto S., Mérour E., Coupanec A.L., Lamoureux A., Bernard J., Brémont M., Millet J.K., Biacchesi S. (2023). Recombinant Viral Hemorrhagic Septicemia Virus with Rearranged Genomes as Vaccine Vectors to Protect against Lethal Betanodavirus Infection. Front. Immunol..

[19] Ma J., Bruce T.J., Jones E.M., Cain K.D. (2019). A Review of Fish Vaccine Development Strategies: Conventional Methods and Modern Biotechnological Approaches. Microorganisms.

[20] Mondal H., Thomas J. (2022). A Review on the Recent Advances and Application of Vaccines against Fish Pathogens in Aquaculture. Aquac. Int..

[21] Reed L.J., Muench H. (1938). A Simple Method of Estimating Fifty per Cent Endpoints. Am. J. Hyg..

[22] Olveira J.G., Souto S., Dopazo C.P., Bandín I. (2013). Isolation of Betanodavirus from Farmed Turbot *Psetta maxima* Showing No Signs of Viral Encephalopathy and Retinopathy. Aquaculture.

[23] Olveira J.G., Souto S., Bandín I., Dopazo C.P. (2021). Development and Validation of a SYBR Green Real Time PCR Protocol for Detection and Quantification of Nervous Necrosis Virus (NNV) Using Different Standards. Animals.

[24] Valero Y., Olveira J.G., López-Vázquez C., Dopazo C.P., Bandín I. (2021). BEI Inactivated Vaccine Induces Innate and Adaptive Responses and Elicits Partial Protection upon Reassortant Betanodavirus Infection in Senegalese Sole. Vaccines.

[25] Souto S., Olveira J.G., López-Vázquez C., Dopazo C.P., Labella A., Bandín I. (2024). Nervous Necrosis Virus (NNV) Vaccination of Carrier Senegalese Sole (*Solea senegalensis*). Aquaculture.

[26] Livak K.J., Schmittgen T.D. (2001). Analysis of Relative Gene Expression Data Using Real-Time Quantitative PCR and the 2-ΔΔCT Method. Methods.

[27] Montero D., Benitez-Dorta V., Caballero M.J., Ponce M., Torrecillas S., Izquierdo M., Zamorano M.J., Manchado M. (2015). Dietary vegetable oils: Effects on the expression of immune-related genes in Senegalese sole (*Solea senegalensis*) intestine. Fish Shellfish Immunol..

[28] Gémez-Mata J., Labella A.M., Bandín I., Borrego J.J., García-Rosado E. (2021). Immunogene expression analysis in betanodavirus infected-Senegalese sole using an OpenArray® platform. Gene.

[29] Venter P.A., Schneemann A. (2008). Recent Insights into the Biology and Biomedical Applications of Flock House Virus. Cell. Mol. Life Sci..

[30] Iwamoto T., Mise K., Takeda A., Okinaka Y., Mori K.I., Arimoto M., Okuno T., Nakai T. (2005). Characterization of Striped Jack Nervous Necrosis Virus Subgenomic RNA3 and Biological Activities of Its Encoded Protein B2. J. Gen. Virol..

[31] Eckerle L.D., Ball L.A. (2002). Replication of the RNA Segments of a Bipartite Viral Genome Is Coordinated by a Transactivating Subgenomic RNA. Virology.

[32] Nishizawa T., Gye H.J., Takami I., Oh M.J. (2012). Potentiality of a Live Vaccine with Nervous Necrosis Virus (NNV) for Sevenband Grouper *Epinephelus septemfasciatus* at a Low Rearing Temperature. Vaccine.

[33] Oh M.J., Gye H.J., Nishizawa T. (2013). Assessment of the Sevenband Grouper *Epinephelus septemfasciatus* with a Live Nervous Necrosis Virus (NNV) Vaccine at Natural Seawater Temperature. Vaccine.

[34] Sudheesh P.S., Cain K.D. (2017). Prospects and Challenges of Developing and Commercializing Immersion Vaccines for Aquaculture. Int. Biol. Rev..

[35] Adams A. (2019). Progress, Challenges and Opportunities in Fish Vaccine Development. Fish Shellfish Immunol..

[36] Péducasse S., Castric J., Thiéry R., Jeffroy J., Ven A.L., Laurencin F.B. (1999). Comparative Study of Viral Encephalopathy and Retinopathy in Juvenile Sea Bass *Dicentrarchus labrax* Infected in Different Ways. Dis. Aquat. Organ..

[37] Collet B. (2014). Innate Immune Responses of Salmonid Fish to Viral Infections. Dev. Comp. Immunol..

[38] Zou J., Secombes C.J. (2011). Teleost Fish Interferons and Their Role in Immunity. Dev. Comp. Immunol..

[39] Medzhitov R. (2007). Recognition of Microorganisms and Activation of the Immune Response. Nature.

[40] Zou J., Secombes C.J. (2016). The Function of Fish Cytokines. Biology.

[41] Ashfaq H., Soliman H., Saleh M., El-Matbouli M. (2019). CD4: A Vital Player in the Teleost Fish Immune System. Vet. Res..

[42] Somamoto T., Koppang E.O., Fischer U. (2014). Antiviral Functions of CD8+ Cytotoxic T Cells in Teleost Fish. Dev. Comp. Immunol..

[43] Zagury D., Burny A., Gallo R.C. (2001). Toward a new generation of vaccines: The anti-cytokine therapeutic vaccines. Proc. Natl. Acad. Sci. USA.

[44] Cheng Y.K., Wu Y.C., Chi S.C. (2017). Humoral and cytokine responses in giant groupers after vaccination and challenge with betanodavirus. Dev. Comp. Immunol..

[45] Chen Y.M., Wang T.Y., Chen T.Y. (2014). Immunity to betanodavirus infections of marine fish. Dev. Comp. Immunol..

[46] Rouxel R.N., Tafalla C., Mérour E., Leal E., Biacchesi S., Brémont M. (2016). Attenuated Infectious Hematopoietic Necrosis Virus with Rearranged Gene Order as Potential Vaccine. J. Virol..

[47] Zeng R., Pan W., Lin Y., He J., Luo Z., Li Z., Weng S., He J., Guo C. (2021). Development of a Gene-Deleted Live Attenuated Candidate Vaccine against Fish Virus (ISKNV) with Low Pathogenicity and High Protection. iScience.

[48] Zeng R., Pan W., Lin Y., Liang M., Fu J., Weng S., He J., Guo C. (2023). A Safe and Efficient Double-Gene-Deleted Live Attenuated Immersion Vaccine to Prevent the Disease Caused by the Infectious Spleen and Kidney Necrosis Virus. J. Virol..

